# Mediastinal Nodal Staging Performance of Combined Endobronchial and Esophageal Endosonography in Lung Cancer Cases: A Systematic Review and Meta-Analysis

**DOI:** 10.3389/fsurg.2022.890993

**Published:** 2022-05-23

**Authors:** Xiaozhen Liu, Kun Yang, Weihong Guo, Muqi Ye, Shaozhong Liu

**Affiliations:** ^1^ Department of Ultrasonography, Zhongshan People's Hospital (ZSPH), Zhongshan, China; ^2^ Department of Respiratory Medicine, Zhongshan People's Hospital (ZSPH), Zhongshan, China

**Keywords:** EBUS, EUS, lung cancer, mediastinal nodal staging, meta-analysis

## Abstract

By searching lliteratures till January 5, 2022, we evaluated the role of the mediastinal nodal staging of endobronchial ultrasound-guided fine-needle aspiration (EBUS) and endoscopic ultrasound-guided fine-needle aspiration (EUS) in lung cancer. A total of 20 studies with 2,961 patients were included in this study. The pooled sensitivity, specificity, PLR, and NLR for EBUS were 0.79, 0.97, 27.29, and 0.25, respectively. EUS showed staging performance similar to EBUS. The staging performance was significantly improved when combining EBUS + EUS.

## Introduction

Around 1.8 million new cases and 1.59 million deaths are recorded every year, making lung carcinoma one of the commonest cancer and the main causes of cancer death among men (1–3). Except for abandon smoking, the best method in decreasing lung cancer incidence and mortality is deemed as the early-stage diagnosis, followed by surgical resection (4).

Accurate staging is the critical step for manageing cases with lung carcinoma. Since the prognosis of cases with lung cancer are affected by the presence of mediastinal lymph node metastasis, lymph node staging is crucial for physicians for clinical diagnosis. In recent years, chest computed tomography (CT) and integrated positron emission tomography (PET)-CT have been extensivly used for nodal staging, and mediastinoscopy is deemed as a “gold standard” for lymph node staging (4–7). Endobronchial ultrasound-guided fine-needle aspiration (EBUS) was implemented since 2004 and was reported to be a less invasive method in diagnosing mediastinal and hilar lymph node metastasis (8). It is compatible with the convex-probe EBUS scope with a diagnostic yield similar to that of mediastinoscopy (9, 10). Similarly, endoscopic ultrasound-guided fine-needle aspiration (EUS) showed mediastinal restaging sensitivity from 75%–92% (11–13). Moreover, aimed to obtain enhanced sensitivity in the initial mediastinal staging of lung cancer, EBUS and EUS are combined due to their complimentary access to mediastinal lymph gland.

In the current study, we aimed to quantitatively analyze the mediastinal nodal staging performance of EBUS and EUS in lung cancer cases.

## Methods

Our current study were done following the preferred reporting items for systematic reviews and meta-analysis (PRISMA) statement (14).

### Literature search

MEDLINE, Cochrane Library, and EMBASE were used for searching targeted studies published up to January 5, 2022. The individual and combined were used to search relevant studies: “mediastinal staging”, “non-small-cell lung cancer”, endoscopic echography”, “fine-needle aspiration”, “EUS”, “EBUS”, “endobronchial ultra-sonography”. The search strategies are described in Supplementary file. The bibliography of on the topic were browsed for obtaining more potential studies.

### Eligibility criteria

Literatures that fulfilled the inclusion criteria were included:

(1) reseachers employed EBUS and EUS(-B) to stage mediastinal lymph nodes in patients with non-small cell lung cancer (NSCLC);
(2) studies reported true positive (TP), true negative (TN), false positive (FP), and false negative (FN);
(3) mediastinal lymph nodes were confirmed by mediastinoscopy, surgical lymph node dissection, or radiological follow-up;
(4) studies published in English;
(5) when the population was reported in duplicate, studies that provided detailed information or were newly published articles were taken into consideration.

Studies were excluded if they were:

(1) short reports, commens, communications, reviews;
(2) papers focused on diagnosing primary lung tumors;
(3) studies included cases after induction therapy;
(4) studies published in other language than English.

### Data extraction and definitions

Needed information were extracted by two authors independently. Each disagreement was settled by consensus. For all included studies, we extracte: name of the first author, the year of publication, countries, study types, sample size, characteristics of cases, characteristics of EBUS and EUS(-B), diagnostic parameters, i.e. TP, TN, FP, and FN. The results were appraised as high/low risk or unclear risk. Moreover, the Grading of Recommendations, Assessment, Development, and Evaluation (GRADE) (15) was applied to evaluate the study evidence quality.

### Quality of studies assessment

We usedthe Quality Assessment of Diagnostic Accuracy Studies 2 (QUADAS-2) tool (16) to assesse the quality of the included studies independently by the two authors. The assessement of QUADAS-2 was based on four items: (1) how the cases were selected; (2) index test, it was the descriptions of how the studies were implented and how the results were interpretatd; (3) reference standard, it includes descriptions standards of the the references; (4) flow and timing, it includeshow the cases were included and excluded.

### Statistical analysis

For each study, a 2 × 2 table was used to assess the test accuracy: sensitivity, specificity, positive like likelihood ratio (PLR), and negative likelihood ratio (NLR). The summary receiver operating characteristic (SROC) curves, the area under the curves (AUCs), and associated standard errors (SEs) were deduced. The sensitivity and specificity from the included studies were pooled by random-effects models and the AUCs of the SROC curves were determined using the DerSimonian-Laird random-effects method. The 95% confidence intervals (CIs) of the merged parameters were used to compare and assess the relative performances of these techniques.

The *I*^2^ was empolyed to evaluate the consistency of the effect size, which assess the variability by percentages. Heterogeneity was described as low, moderate, and high according to the values of *I*^2^ as 25%, 50%, and 75%, respectively (17). We also included QUADAS-2 score, study design, type of confirmation, and the country as covariates of univariate meta-regression analysis (weighted inverse variance). The relative DOR (RDOR) was calculated. Publication biases were calculated by Begg’s rank correlation (18) and Egger’s weighted regression methods (19). We used Meta-Disc software programs (version 1.4, Ramón y Cajal Hospital, Madrid, Spain) and R programme (version 4.2.0) for the data analyses. Publication biases were carried out using STATA 15.0. *p *< 0.05 indicated statistical significance for all analyses.

### Role of the funding source

No external funding was received for this study. The corresponding author had full access to all the data and made the final decision of publication.

## Results

### Study selection

**Figure 1:**
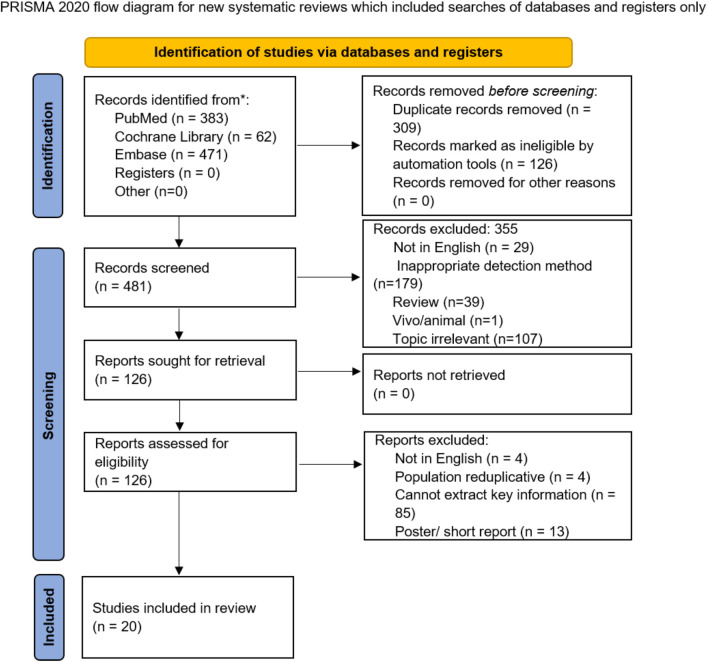
Flow chart of the study selection process.

The search strategy obtained 916 potentially relevant studies and 309 of them were excluded due to overlap, while 355 were excluded after screening the titles or abstracts due to topic relevance or improper study design. Finally, 20 articles (20–39) were included. The study selection is illustrated in Figure 1.

### Study characteristics

Table 1 presents the characteristics of study participants. A total of 20 studies with 2,961 participants were included in this meta-analysis. The sample size of the studies ranged from 20 to 696, and the studies were published between 2005 and 2019. Among these, 5 studies were conducted in South Korea, 3 each in the USA, Netherlands, and Poland, 2 in Japan, and 1 each in the UK, Denmark, Germany, and Turkey. Moreover, 8 studies assessed the diagnostic performance using contrast-enhanced (CE)-CT while 4 used CE-magnetic resonance imaging (MRI). The majority of the studies were prospective, and one was a randomized controlled trial. The data of each study are presented in Table 2.

**Table 1:** Characteristics of the included studies.

Study	Country	Study design	Age (years)	Males (*n*)	Type of sedation	Reference standard	NSCLC (*n*)	SCLC (*n*)	Other (*n*)
Rintoul et al. 2005	UK	Prospective	65 (45–86)	10	Conscious sedation	Surgery and follow-up	9	11	0
Vilmann et al. 2005	Denmark	Prospective	61	23	Conscious sedation	Surgery and follow-up	20	13	0
Wallace et al. 2008	USA	NA	69 (60–76)	66	Conscious sedation	Surgery and follow-up	13	16	0
Herth et al. 2010	Germany	NA	57.6	83	Conscious sedation	Surgery and follow-up	619	0	0
Hwangbo et al. 2010	Korea	Prospective	64.5 (34–80)	113	Conscious sedation	Surgery	149	1	0
Annema et al. 2010	Netherlands	Prospective	65	99	Moderate sedation	Surgery	123	0	0
Szlubowski et al. 2010	Poland	Prospective	61.8 ± 8.4	94	Local anaesthesia and intravenous sedation	Surgery	120	0	0
Ohnishi et al. 2011	Japan	Prospective	69 (40–85)	79	Conscious sedation	Surgery	NA	NA	NA
Liberman et al. 2014	USA	Prospective	64 ± 9.4	82	Conscious sedation	Surgery	166	0	0
Szlubowski et al. 2015	Poland	Prospective	NA	150	Mild sedation	Surgery	NA	NA	NA
Kang et al. 2014	Korea	RCT	63.21 ± 7.91/62.94 ± 8.39	120	Conscious sedation	Surgery	151	3	6
Oki et al. 2014	Japan	Prospective	68.3 ± 8.6	103	Conscious sedation	Surgery and follow-up	146	2	2
Hauer et al. 2015	Poland	Prospective	65 (30–84)	367	NA	Surgery	673	4	19
Jhun et al. 2012	Korea	Prospective	65 (31–82)	117	Conscious sedation	Surgery	151	0	0
Lee et al. 2014	Korea	NA	66.0 (43–86)	36	Conscious sedation	Surgery	39	3	2
Dooms et al. 2015	Netherlands	Prospective	65 ± 9.8	NA	Moderate sedation	Surgery	100		
Um et al. 2015	Korea	Prospective	62 (34–76)	117	Conscious sedation	Surgery	130	0	8
Vial et al. 2018	USA	Prospective	66.3 ± 9.6	37	Moderate sedation	Surgery	75	0	0
Crombag et al. 2019	Netherlands	Prospective	67 ± 8.9	148	Moderate or deep sedation	Surgery	208	11	6
Tutar et al. 2018	Turkey	Prospective	NA	NA	Conscious sedation	Surgery	20	0	0

*Abbreviations: EBUS, endobronchial endoscopy; EUS, oesophageal endoscopy; NSCLC, non-small-cell lung cancer; SCLC, small cell lung cancer; RCT, randomized controlled trial; NA, not available.*

**Table 2:** Accuracy in detecting mediastinal nodal metastases across included studies

Study	N	EBUS				EUS				EBUS + EUS			
		TP	FP	FN	TN	TP	FP	FN	TN	TP	FP	FN	TN
Rintoul et al. 2005	18	11	0	2	5	3	0	1	2	11	0	2	5
Vilmann et al.2005	31	NA	NA	NA	NA	NA	NA	NA	NA	20	0	0	11
Wallace et al. 2008	138	29	0	13	96	29	0	13	96	39	0	3	96
Herth et al. 2010	139	65	0	6	68	63	0	8	68	68	0	3	68
Hwangbo et al. 2010	143	38	0	7	98	NA	NA	NA	NA	41	0	4	98
Annema et al. 2010	123	NA	NA	NA	NA	NA	NA	NA	NA	58	0	13	52
Szlubowski et al. 2010	120	13	1	15	99	14	1	14	99	19	2	9	90
Ohnishi et al. 2011	110	25	0	14	71	19	0	20	71	28	0	11	71
Liberman et al. 2014	166	39	0	15	112	33	0	21	112	49	0	5	112
Szlubowski et al. 2015	214	53	3	9	43	61	1	5	54	NA	NA	NA	NA
Kang et al. 2014	160	29	5	5	40	23	5	2	49	NA	NA	NA	NA
Oki et al. 2014	150	17	0	16	113	15	0	18	113	24	0	8	113
Hauer et al. 2015	696	NA	NA	NA	NA	162	5	54	475	NA	NA	NA	NA
Jhun et al. 2012	151	142	1	13	70	NA	NA	NA	NA	NA	NA	NA	NA
Lee et al. 2014	44	23	0	6	8	NA	NA	NA	NA	29	0	0	8
Dooms et al. 2015	100	9	13	15	63	NA	NA	NA	NA	18	10	6	66
Um et al. 2015	138	66	0	9	52	61	0	14	52	NA	NA	NA	NA
Vial et al. 2018	75	5	16	4	50	NA	NA	NA	NA	NA	NA	NA	NA
Crombag et al. 2019	225	79	0	24	122	NA	NA	NA	NA	84	0	19	122
Tutar et al. 2018	20	9	0	2	9	9	0	2	6	10	0	1	9

*Abbreviations: EBUS, endobronchial endoscopy; EUS, oesophageal endoscopy; TP, true positive; TN, true negative; FP, false positive; FN, false negative; NA, not available.*

### Assessment of study quality and risk of bias

None of the included studies was judged as high risk, suggesting that the quality of all the eligible articles was acceptable. The quality assessment results are presented in Supplementary Table S1.

### Staging accuracy of EBUS

**Figure 2:**
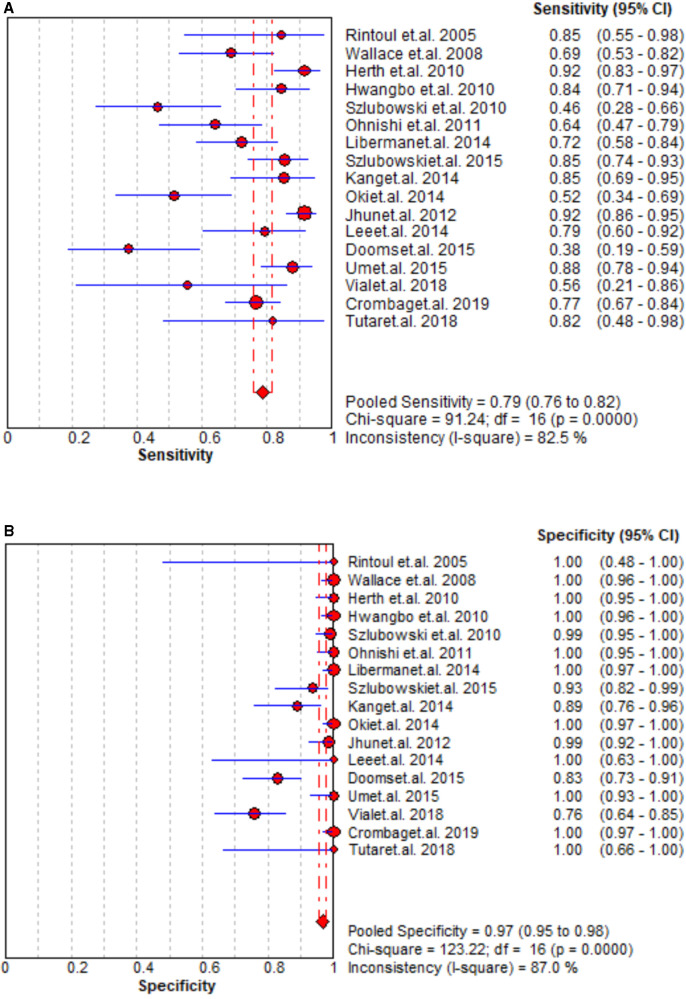
Summary of pooled sensitivity and specificity of EBUS.

The staging performance of EBUS of 17 studies revealed sensitivity and specificity 0.46–0.92 and 0.76–1.00, respectively. The pooled sensitivity, specificity, PLR, and NLR for EBUS for mediastinal nodal staging in lung cancer were 0.79 (95% CI = 0.76–0.82), 0.97 (95% CI = 0.95–0.98), 27.29 (95% CI = 9.82–75.83), and 0.25 (95% CI = 0.18–0.36), respectively. The results are presented in Figure 2 and Supplementary Figure S1. As shown in Supplementary Figure S2, the pooled AUC for EBUS was 0.895 ± 0.0594 SE).

### Staging accuracy of EUS

**Figure 3:**
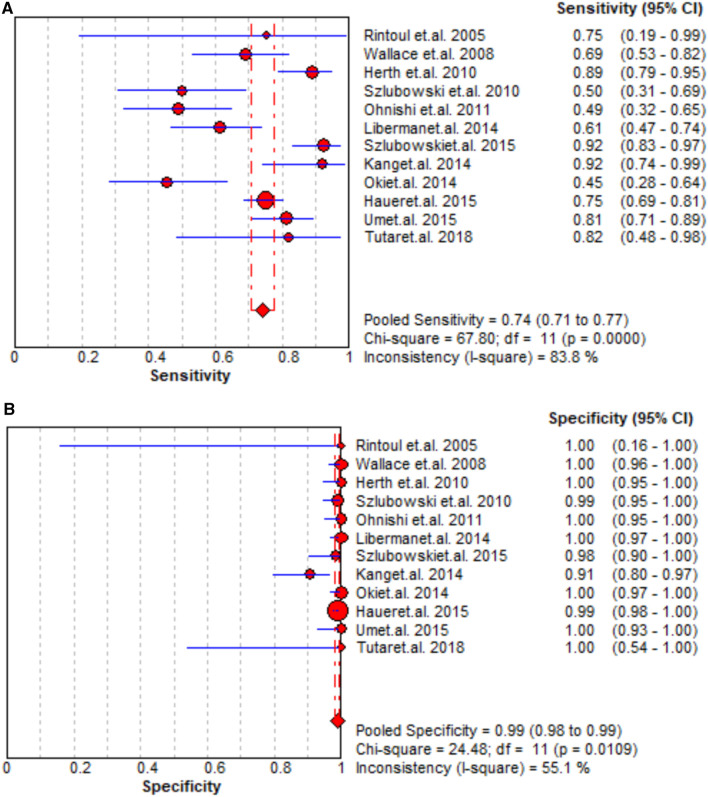
Summary of pooled sensitivity and specificity of EUS.

A total of 12 studies reported that the staging performance of EUS was similar to that of EBUS with sensitivity and specificity 0.49–0.92 and 0.98–1.00, respectively. When pooling the results, the combined sensitivity, specificity, PLR, and NLR were 0.74 (95% CI = 0.71–0.77), 0.99 (95% CI = 0.98–0.99), 36.91 (95% CI = 16.73–81.40), and 0.28 (95% CI = 0.20–0.39), respectively, but the AUC was slightly high as 0.9682 ± 0.0143. The data of sensitivity and specificity, PLR and NLR, and AUCs are presented in Figures 3, Supplementary Figures S3 and S4, respectively.

### Staging accuracy of EBUS + EUS

**Figure 4:**
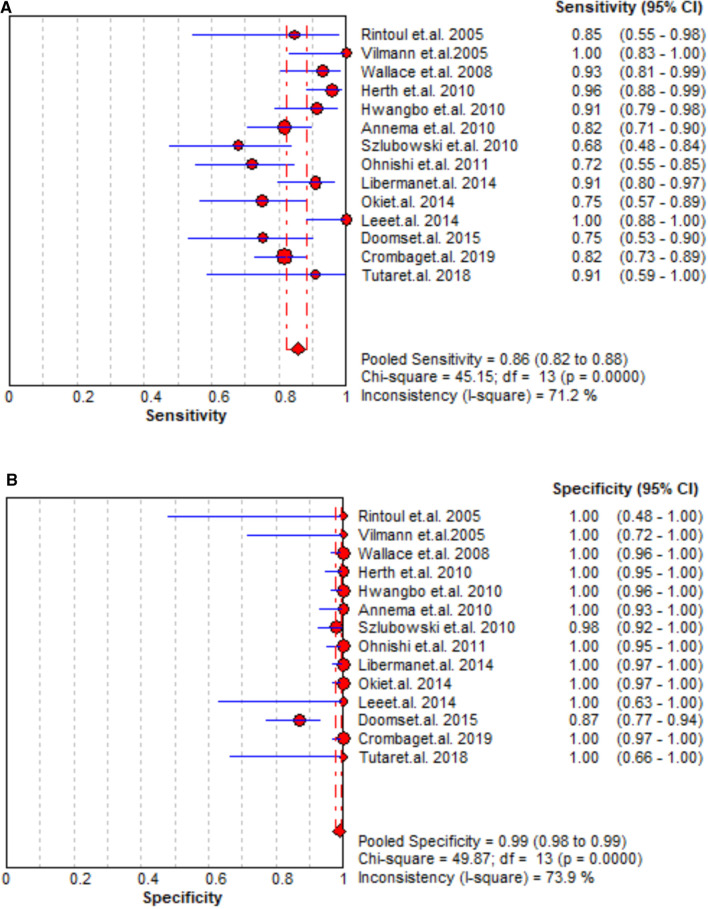
Summary of pooled sensitivity and specificity of the combination of EBUS and EUS.

A total of 14 studies assessed the staging accuracy of EBUS + EUS, and the majority of them reported significantly high sensitivity (0.72–0.96). A significantly improved staging accuracy of EBUS + EUS was observed with pooled sensitivity, specificity, PLR, and NLR as 0.86 (95% CI = 0.82–0.88, Figure 4), 0.99 (95% CI = 0.98–0.99, Figure 4), 49.48 (95% CI = 15.17–161.35, Supplementary Figure S5), and 0.17 (95% CI = 0.12–0.23, Supplementary Figure S5), respectively, and AUC was also high (0.0.9722 ± 0.0194; Supplementary Figure S6).

### Multiple regression analysis

As shown in Table 3, studies with high-quality QUADAS-2 score, varied design, conducted in various countries, and confirmed by different methods indicated that these factors do not affect the staging accuracy substantially.

**Table 3:** Weighted meta-regression of the effects of methodologic al characteristics, study design, country, and type of confirmation.

Covariates	Coeff.	RDOR	*p* value
**EBUS**			
QUADAS-2	−0.075	0.93	0.86
Type of confirmation	−0.457	0.63	0.76
Study design	−0.505	0.60	0.73
Country	0.010	1.01	0.95
**EUS**			
QUADAS-2	0.054	1.06	0.76
Type of confirmation	0.890	2.44	0.43
Study design	−1.307	0.27	0.23
Country	−0.058	0.94	0.61
**EBUS + EUS**			
QUADAS-2	0.217	1.24	0.50
Type of confirmation	−0.467	0.63	0.70
Study design	−2.469	0.08	0.12
Country	−0.010	0.99	0.94

*Abbreviations: EBUS, endobronchial endoscopy; EUS, oesophageal endoscopy; QUADAS-2, the Quality Assessment of Diagnostic Accuracy Studies 2.*

### Certainty of the evidence

Overall, the certainty of the evidence was low when assessed according to GRADE criteria.

### Publication bias

No significant publication bias was seen with *P*-values more than 0.05of Begg’s rank correlation analysis and Egger’s weighted regression analysis (Supplementary Table S2).

## Discussion

In our current meta-analysis, the accuracy of EBUS and EUS was investigated for the mediastinal nodal staging in lung cancer cases. 20 studies with 2,961 participants were finally included and pooled. Both EBUS and EUS provide accurate performance in the mediastinal nodal staging in lung cancer cases. When combining EBUS and EUS, the staging performance was improved further.

The staging of lung cancer started with radiology (19). F-Fluorodeoxyglucose PET or PET-CT reported a high diagnostic accuracy for mediastinal staging with sensitivity and specificity as 0.85 and 0.90, respectively (40). A previous meta-analysis reported that the sensitivity and specificity of PET-CT for detecting metastatic lymph nodes was 0.78 and 1.00, respectively (41). Similar results were also reported by previous meta-analyses (42–45). In the current study, with inclusion of more revelant studies, the sensitivity and specificity was 0.79 and 0.97 for EBUS and 0.74 and 0.99 for EUS, respectively; the staging performance in detecting metastatic lymph nodes was similar to that of PET-CT. Although EBUS provides access to mediastinal lymph nodes commonly involved in lung cancer, and EUS complements this by accessing nodes beyond the reach of EBUS in the inferior mediastinum, the majority of the lymph node stations in the mediastinum are accessed by endosonographic guidance (46, 47). Therefore, the enhanced staging performance of the combination of EBUS and EUS is reasonable and undisputed.

However, the finding in the current study showed that EBUS and EUS were similar to PET, but it does not propagate that it should be adopted as the preferred choice for the staging of mediastinal lymph nodes in patients with known or suspected lung cancer (48, 49). Unlike CT or PET-CT, EBUS or EUS acquires invasive tissue samples. However, EBUS has the advantage of assessing the lymph node stations that are in close proximity to the airways, such as paratracheal and subcarinal stations (50, 51). The non-invasive restaging technique, i.e., CT or PET-CT, suggested persistent mediastinal lymph node involvement post-induction therapy that requires tissue confirmation (52, 53), and EBUS or EUS provided the best access to those lymph node stations. In previous years, the obstruction for endosonography-guided needle sampling techniques, the EBUS or EUS, has limited accuracy in the mediastinal restaging of lung cancer (54, 55). These findings highlighted the clinical meaning of EBUS or EUS. Nevertheless, due to the performance of EBUS or EUS is highly effected by clinical experiences of operators. Establishing EBUS or EUS as a diagnostic and therapeutic method is therefore still a challengd (56). Given the cost of interventional EBUS or EUS is high and EBUS or EUS is deficient in majority of regions, tsherefore, when these two methods are implemented, several concerns arise, such as they are likely to be more operator-dependent and have a steeper learning curve compared to the initial mediastinal staging for lung cancer cases (57, 58).

Nevertheless, the current study has some limitations when interpreting the results. First, 20 studies with limited lung cancer cases were included in the current meta-analysis. Due to liminted included cases, we canot conduct the subgroup or sensitivity analyses according to the stages of lung cancer and the type of carcinoma. Second, the mean age and sex ratio of the participants in each study varied largely, which in turn caused heterogeneity and decreased the stability of the results. Third, the stages of lung cancer differed among the included studies, which might decrease the comparability of the included studies. Fourth, the heterogeneity of the imaging technique was moderate, and the results require further validation in the future. However, due to the limited number of included studies, subgroup analysis on the imaging technique could not be performed. However, we cannot ensure that all parameters are constant. Fifth, the paper was registered in retrospectives in the PROSPERO (ID: 323791), which decrease transparency of the meta-analyses. Sixth, potential language bias might exist as articles not published in English were excluded.

## Conclusions

In conclusion, both EBUS and EUS provide accurate and comparable staging performance. The combination EBUS and EUS provide enhanced accurate results on the staging of lung cancer cases. However, additional studies with large sample sizes are warranted to further confirm our findings.
