# Supplementary material for: Mediastinal Nodal Staging Performance of Combined Endobronchial and Esophageal Endosonography in Lung Cancer Cases: A Systematic Review and Meta-Analysis
Source: Front Surg. 2022 May 23;9:890993. doi: 10.3389/fsurg.2022.890993 (PMC9168235; doi:10.3389/fsurg.2022.890993)
Supplement: Supplementary file 1 [file Table_1_v1.docx]

1. **Search strategies**

**PubMed: 383 Results, 1.5.2022**

"Mediastinum"[MeSH Terms] AND (("Endosonography"[MeSH Terms] OR ("Ultrasonography"[MeSH Terms] OR "Ultrasonics"[MeSH Terms])) AND "Lung Neoplasms"[MeSH Terms])

**Cochrane Library: 62 Results, 1.5.2022**

MeSH descriptor: [Lung Neoplasms] explode all trees

MeSH descriptor: [Endosonography] explode all trees

MeSH descriptor: [Ultrasonography] explode all trees

**Embase: 471 Results, 1.5.2022**

'Lung Neoplasms'/exp

'Endosonography'/exp

'Ultrasonography'/exp

# Supplementary Figures and Tables

**Supplementary Table 1. Quality assessment of included studies by QUADAS-2**

| **Study** | **Patient Selection (3)** | **Index Test (2)** | **Reference Standard (2)** | **Flow and Timing (4)** | **Total quality scores** |
| --- | --- | --- | --- | --- | --- |
| Rintoul et.al. 2005 | 3 | 2 | 2 | 3 | 10 |
| Vilmann et.al.2005 | 3 | 2 | 2 | 4 | 11 |
| Wallace et.al. 2008 | 3 | 2 | 2 | 4 | 11 |
| Herth et.al. 2010 | 2 | 2 | 2 | 3 | 9 |
| Hwangbo et.al. 2010 | 2 | 2 | 2 | 3 | 9 |
| Annema et.al. 2010 | 2 | 2 | 2 | 4 | 10 |
| Szlubowski et.al. 2010 | 2 | 1 | 2 | 2 | 7 |
| Ohnishi et.al. 2011 | 3 | 2 | 2 | 3 | 10 |
| Libermanet.al. 2014 | 3 | 2 | 2 | 4 | 11 |
| Szlubowskiet.al. 2015 | 3 | 2 | 2 | 3 | 10 |
| Kanget.al. 2014 | 2 | 2 | 2 | 2 | 8 |
| Okiet.al. 2014 | 3 | 2 | 2 | 3 | 10 |
| Haueret.al. 2015 | 2 | 1 | 1 | 3 | 7 |
| Jhunet.al. 2012 | 2 | 2 | 2 | 3 | 9 |
| Leeet.al. 2014 | 3 | 2 | 2 | 3 | 10 |
| Doomset.al. 2015 | 3 | 2 | 2 | 4 | 11 |
| Umet.al. 2015 | 3 | 2 | 2 | 3 | 10 |
| Vialet.al. 2018 | 2 | 2 | 2 | 3 | 9 |
| Crombaget.al. 2019 | 3 | 2 | 2 | 4 | 10 |
| Tutaret.al. 2018 | 3 | 2 | 2 | 3 | 10 |

**Supplementary Table 2. Publication bias and heterogeneity of summarized outcomes**

| **Outcomes** | **Publication bias** | |
| --- | --- | --- |
|  | **Begg (*P* value)** | **Egger (*P* value)** |
| Summarized staging accuracy of EBUS | 0.19 | 0.15 |
| Summarized staging accuracy of EUS | 0.41 | 0.39 |
| Summarized staging accuracy of EBUS+EUS | 0.71 | 0.84 |


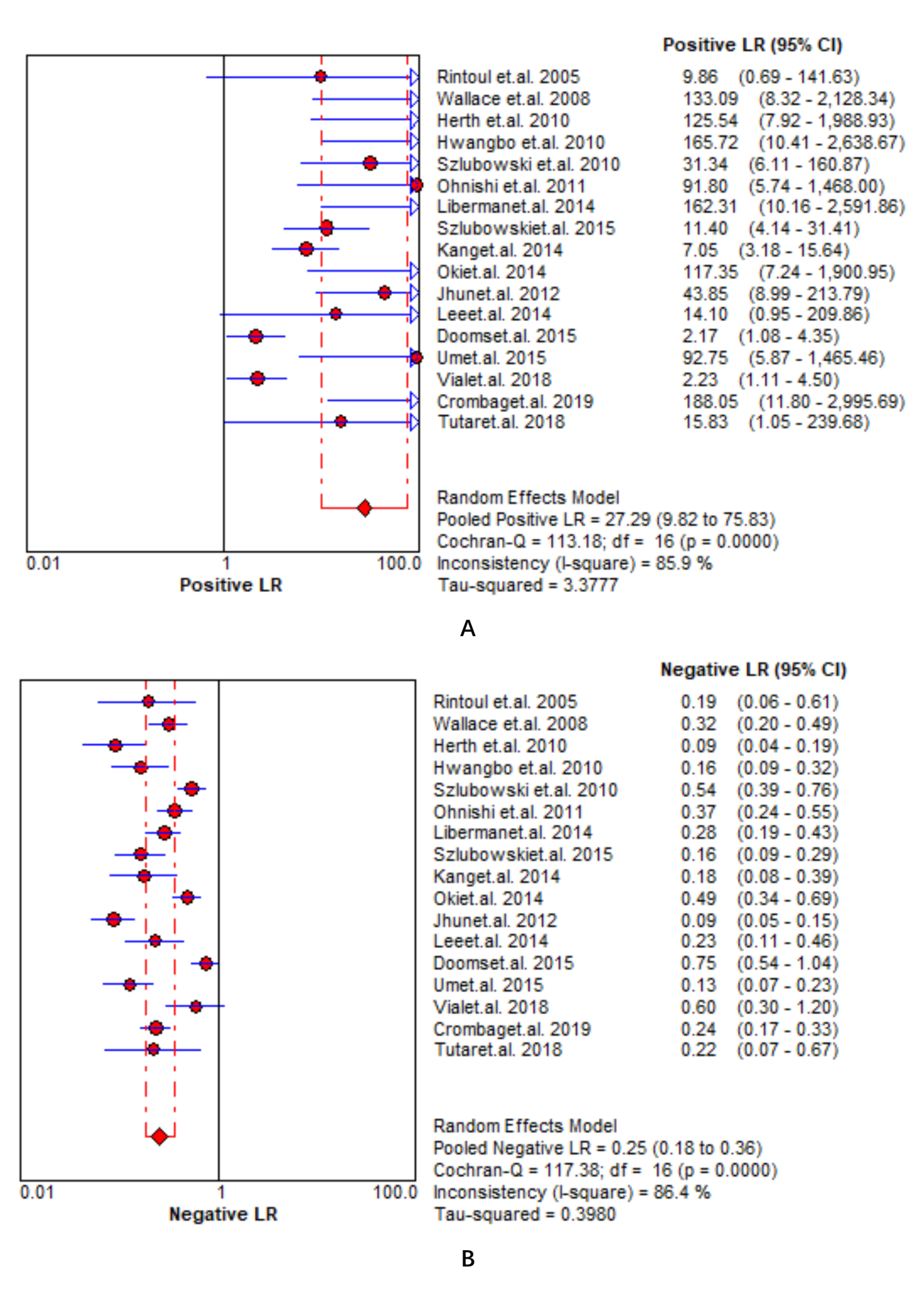


**Figure S1.** Summary of pooled PLR and NLR of EBUS.


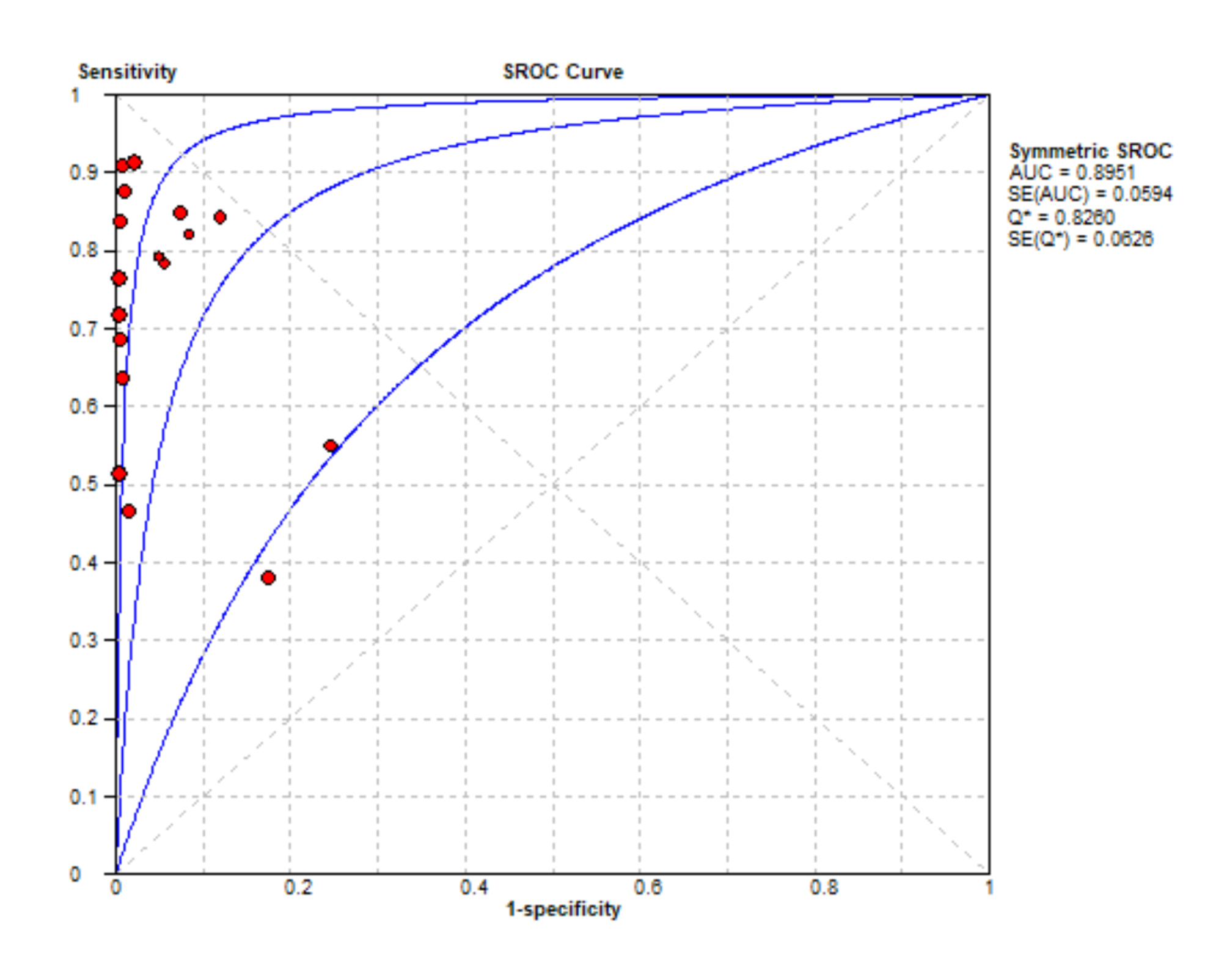


**Figure S2.** Summary of pooled AUC of EBUS.


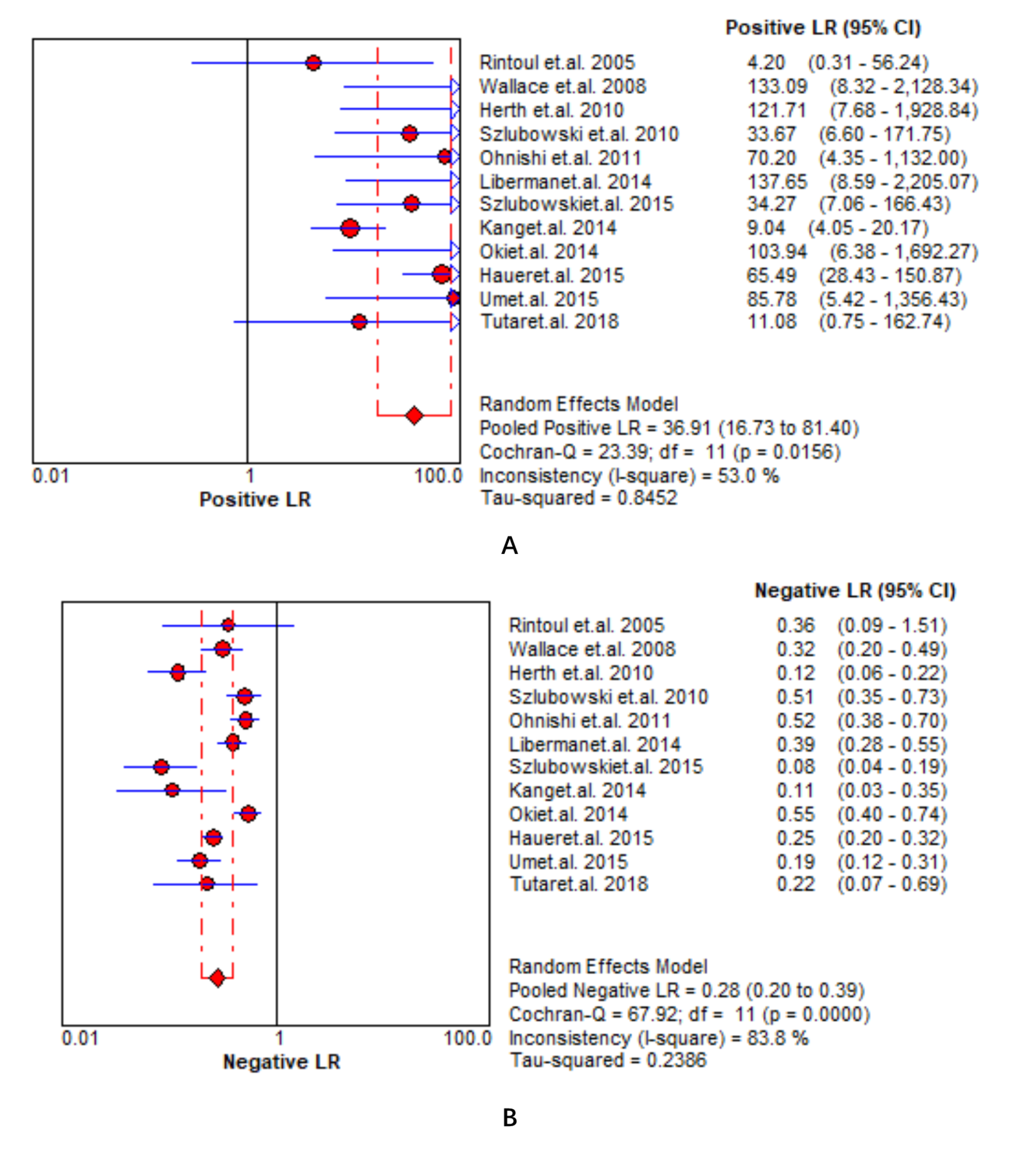


**Figure S3.** Summary of pooled PLR and NLR of EUS.


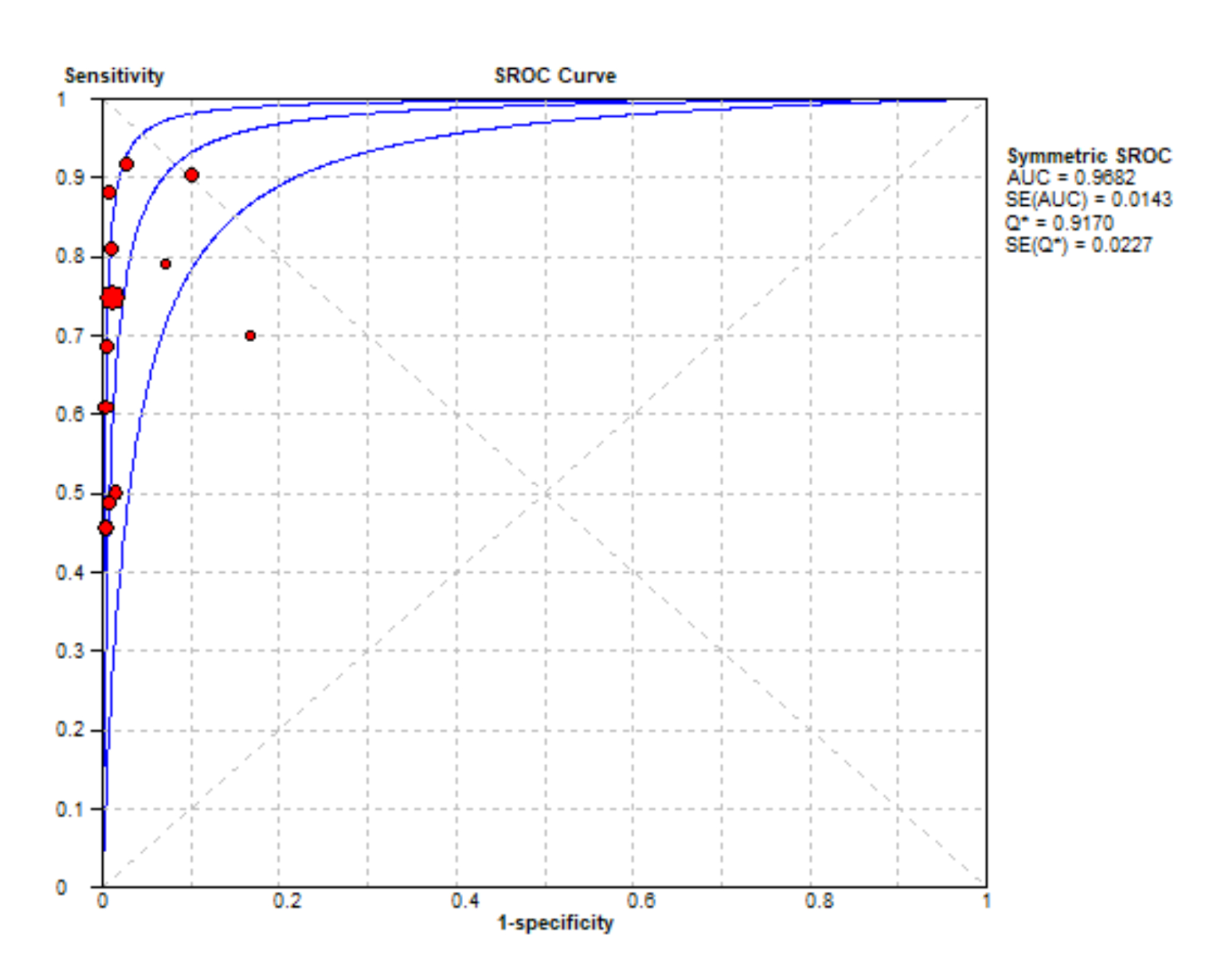


**Figure S4.** Summary of pooled AUC of EUS.


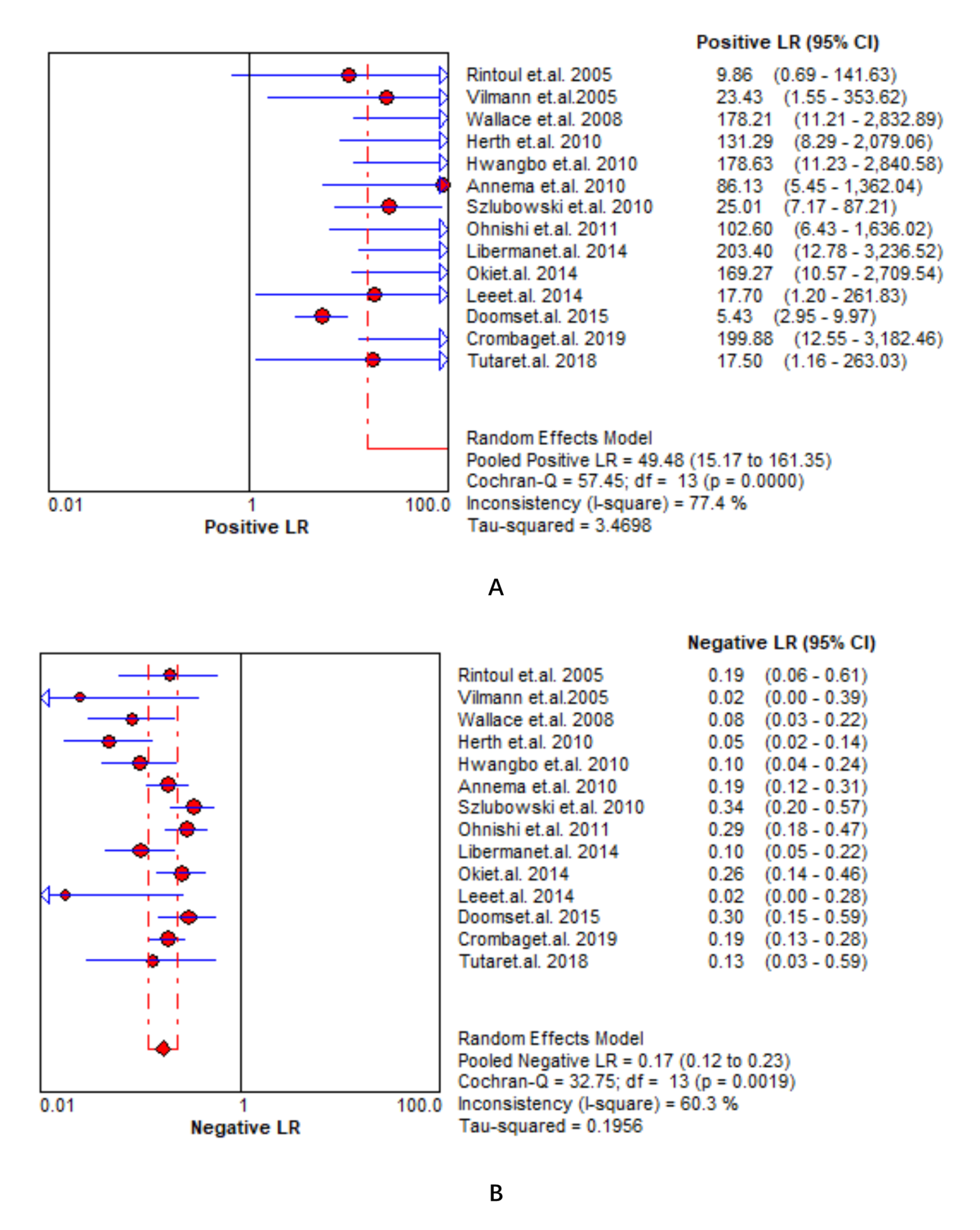


**Figure S5.** Summary of pooled PLR and NLR of EBUS and EUS.


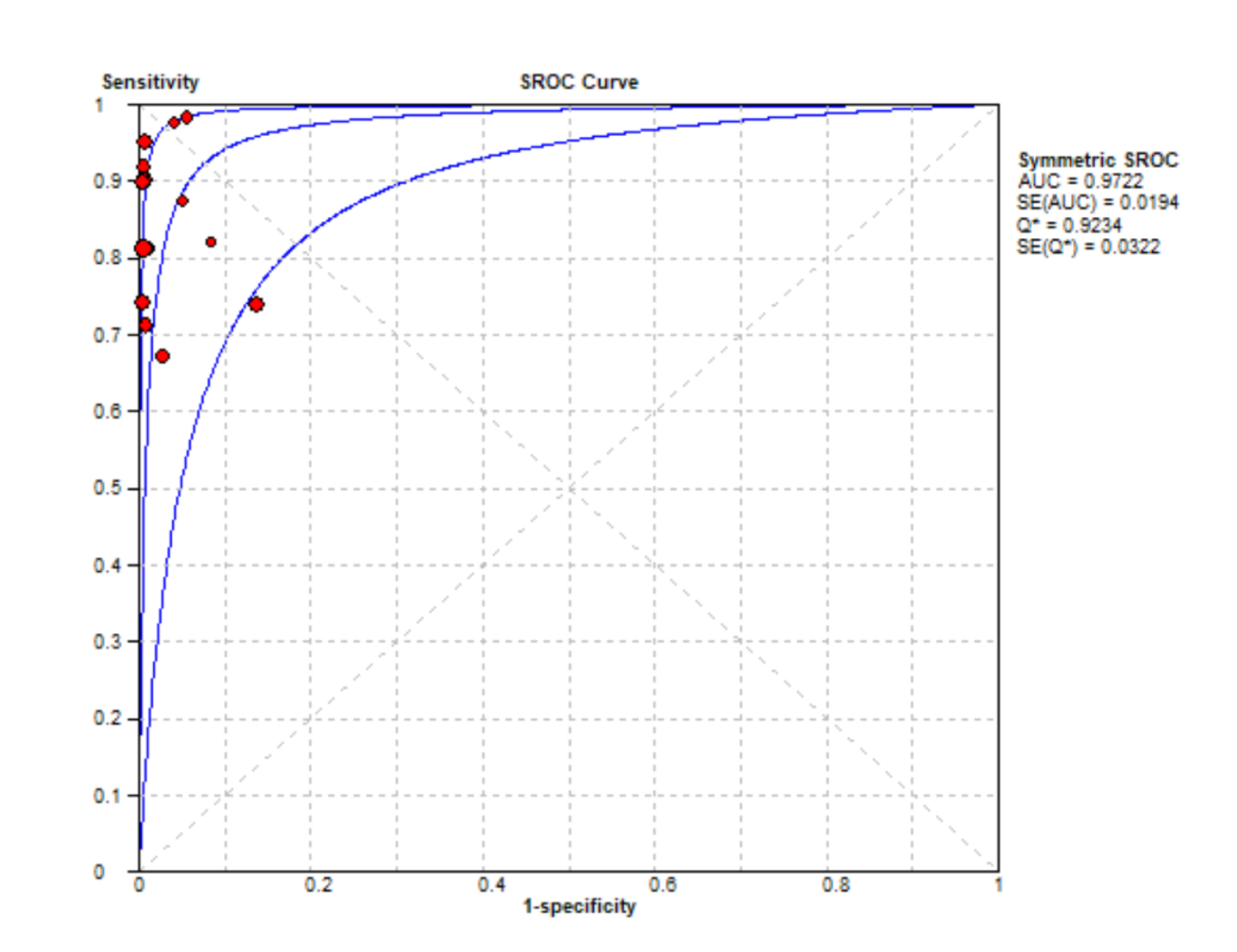


**Figure S6.** Summary of pooled AUC of EBUS and EUS.
